# Editorial: Quality of life in young cochlear implant recipients: Are there controlling factors and regional differences?

**DOI:** 10.3389/fpsyg.2022.1109242

**Published:** 2022-12-15

**Authors:** Maria Huber, Hyo-Jeong Lee, Margreet Langereis, Anneke Vermeulen

**Affiliations:** ^1^Department of Otorhinolaryngology, Paracelsus Medical University, Salzburg, Austria; ^2^Department of Otolaryngology, Hallym University Medical Center, Chuncheon, South Korea; ^3^Research Department, Pento Speech and Hearing Centres, Nijmegen, Netherlands

**Keywords:** quality of life, children and adolescents with hearing loss, cochlear implants, regional differences, controlling factors

Severe and profound childhood hearing loss is a medical condition that can affect the functional development of the brain (Manno et al., 2021; Calmels et al., 2022; Grégoire et al., 2022) and quality of life (QoL). Untreated childhood hearing loss can have consequences beyond the acquisition of spoken language (Blamey et al., 2001; Anne et al., 2017). Not only communication and social interaction (Lieu et al., 2020), as well as self-image (Theunissen et al., 2014), can be affected, but also cognition (Martínez-Cruz et al., 2009) and school performance (Lieu, 2004; Antia et al., 2009), with possible adverse consequences for the QoL (Roland et al., 2016; Lieu et al., 2020). Therefore, the rehabilitation of children with hearing loss aims to restore hearing ability and optimize their developmental potential to enhance their QoL.

A cochlear implant (CI) is an electronic medical device that provides auditory access to speech sounds that cannot be supplied by sound amplification through conventional hearing aids in individuals with severe and profound hearing loss. Early application of CI facilitates spoken language acquisition (Percy-Smith et al., 2008; Peters et al., 2010; Kronenberger et al., 2020; Sharma et al., 2020; Romano et al., 2021; Boerrigter et al., 2022) and promotes participation in mainstream education (Huber et al., 2008, 2014; Huber and Kipman, 2012; Sarant et al., 2015). In addition, CIs seem conducive to the psychosocial prerequisites (e.g., empathy) for social participation (Sarant et al., 2015; Boerrigter et al., 2019, 2021; Tsou et al., 2021). In children, the use of CI can at least partly reverse the effects of hearing loss on the brain (Lee et al., 2020; Lieu et al., 2020; Sharma et al., 2020; Wang et al., 2021).

Several studies reported positive correlations between these CI-specific benefits and the QoL of children and adolescents with hearing loss, such as speech recognition (especially in noisy environments) and spoken language skills (Huber, 2005; Haukedal et al., 2018, 2020; Suneel et al., 2020; Ching et al., 2021), and academic achievement (Van der Straaten et al., 2020).

“Quality of life” refers to different aspects of a person's life, such as economic status, rights, culture, and health (Fayed et al., 2012) with “health-related quality of life” or HRQoL being commonly regarded as a sub-domain of the more global concept of QoL [World Health Organization (WHO), 1948; Davis et al., 2006]. According to the well-validated model of Wilson and Cleary (1995), HRQoL results from biological/physiological variables, symptom status, functional status, and subjective perception of one's state of health (Bakas et al., 2012; Ojelabi et al., 2017).

The present small volume in Frontiers in Psychology, section auditory cognitive neuroscience provides an overview of the state-of-the-art of different pertinent aspects of QoL in young CI recipients. We were particularly interested in high-quality papers that addressed the Research Topics of behavioral and neural correlates and regional differences in QoL, for example, due to societal, cultural, and ethnic differences.

The retrospective study of Warner-Czyz et al. “compared the parent-reported cochlear implant-specific quality of life summary data across 14 published studies spanning 11 countries and nine languages.” Across countries, social and communicative interaction abilities were appraised most positively. The largest differences were found in the communication domain. The authors assumed that limited access to cochlear implantation and rehabilitation, cultural differences in awareness of hearing loss, and differing expectations might explain these differences in parental ratings on the QoL.

The technical progress of cochlear implants is beneficial but also may have limitations. Huber's perspective paper addresses the possible impact of some CI risks listed in the American Food and Drug Administration (FDA)^1^ of pediatric cochlear implantation on the QOL. From this list, medical and device-related complications, lifelong dependency on the implanted device, and neurosecurity risks (CI technology is an interface technology) may be particularly relevant for young CI users. The author suggested that the mere possibility of device failure, peer victimization due to the device the person will depend on for life, or cybersecurity breaches may already have a negative impact on QoL. However, as the author acknowledges, studies are needed to examine these assumptions.

The qualitative study of Rijke et al. informs about the experience of Dutch adolescents and young adults with CIs and with conventional hearing aids. The participants reported that they could participate in hearing society; however, they reported challenges such as dependency on the technical device (compare Huber) and feeling often misunderstood and sometimes stigmatized when comparing themselves to typical-hearing peers.

The perspective paper of Schweinberger and von Eiff points to the importance of new methods for training vocal emotion recognition, morphing and caricaturing. These methods use “digitally modified stimuli with extended diagnostic information” (Schweinberger and von Eiff). The authors suggested that this training will have a comprehensive positive impact on the QoL of children with CI. From a socio-emotional point of view, recognizing the emotional timbre of the other person's voice is likely to be of great importance for young CI users.

## Concluding remarks

This small volume provides some novel insights in QoL of young CI recipients. Improved communication, including vocal emotion recognition, and social participation seem to be important factors for a good quality of life in many cultures. There seem to be cultural differences in how hearing loss in childhood is perceived and experienced and how it affects QoL, at least from the perspective of parents. In addition, it seems to be important to be aware of device-specific (CI) risks in the context of QoL.

However, there are also limitations. Not all regions have access to cochlear implants and rehabilitation that is affordable for all people. This can make it difficult to compare studies addressing QoL of young individuals with CI. Furthermore, two out of four contributions provide information on perspectives. Further studies are needed.

So, the papers in this small volume raise more questions than answers. Studies that provide possible answers to these questions would come from a wide variety of disciplines, including clinical psychology, educational science, audiology, otology-neurotology, neuroscience, computer science, electrical engineering, and sociology.

## Author contributions

All authors listed have made a substantial, direct, and intellectual contribution to the work and approved it for publication.

## Footnotes

^1^FDA https://www.fda.gov/medical-devices/cochlear-implants/benefits-and-risks-cochlear-implants.
